# Supplementary material for: Influence of LDL-Cholesterol Lowering on Coronary Plaque Progression of Non-Target Lesions in Patients Undergoing Percutaneous Coronary Intervention: Findings from a Retrospective Study
Source: J Clin Med. 2023 Jan 18;12(3):785. doi: 10.3390/jcm12030785 (PMC9917377; doi:10.3390/jcm12030785)
Supplement: Supplementary file 1 [file jcm-12-00785-s001.zip › jcm-2123299-supplementary.pdf]

Table S1. Subgroup analysis according to follow-up LDL-C levels.

**Variables**	**Non-optimal Subgroup A (LDL ≥ 1.4 mmol/L and LDL-C Reduction < 50%，*n* = 643)**	**Non-Optimal Subgroup B (LDL ≥ 1.4 mmol/L and LDL-C Reduction ≥ 50%，*n* = 39)**	**Non-Optimal Subgroup C (LDL-C < 1.4 mmol/L and LDL-C reduction < 50%，*n* = 111)**	**Optimal Group (LDL-C < 1.4 mmol/L and LDL-C Reduction ≥ 50%，*n* = 54)**
**Baseline characteristics**				
Age (years)	62.91±11.14	60.28±10.81	63.33±11.10	65.84±10.89
Male (%)	478 (75.34)	27 (69.23)	94 (84.68)*	40 (74.07)
History of hypertension (%)	436 (67.81)	20 (51.28)	80 (72.07)	34 (62.96)
History of DM (%)	372 (57.85)	23 (58.97)	72 (64.86)	35 (64.81)
History of dyslipidemia (%)	127 (19.75)	8 (20.51)	15 (13.51)	8 (14.81)
History of CKD (%)	15 (2.33)	1 (2.53)	3 (2.70)	0 (0.00)*
History of ACS (%)	110 (17.11)	6 (15.38)	21 (18.92)	11 (20.37)
FPG (mmol/L)	5.62±2.91	5.03±3.72	5.77±3.00	6.04±2.40
HbA1C (%)	4.09±3.28	3.81±3.80	4.46±3.12	4.79±3.25
TG (mmol/L)	1.76±1.07	1.70±1.19	2.07±2.33	1.75±1.79
TC (mmol/L)	4.12±1.30	5.80±2.25	3.28±0.83	4.05±1.62
HDL-C (mmol/L)	1.00±0.32	1.09±0.34	0.97±0.30	0.98±0.42
LDL-C (mmol/L)	2.49±0.82	3.99±0.89*	1.59±0.49*	2.84±0.94*
SCr (mmol/L)	81.56±50.75	70.01±25.38	79.96±20.03	74.72±30.43
UA (mmol/L)	328.88±105.69	308.08±103.06	334.17±103.46	318.84±123.72
Follow-up characteristics				
CAG Interval (days)	450.60±99.95	442.41±99.22	433.03±106.57	468.35±109.38
FPG (mmol/L)	5.78±2.72	5.74±3.08	5.98±2.55	6.03±2.64
HbA1C (%)	4.35±3.34	3.79±3.78	4.94±3.09	4.45±3.21
TG (mmol/L)	1.66±0.95	1.31±1.02*	1.61±1.25	1.52±1.17
TC (mmol/L)	3.82±0.97	3.31±0.43*	2.81±0.50*	2.77±0.59*
HDL-C (mmol/L)	1.07±0.28	1.08±0.22	1.04±0.29	1.52±0.28
LDL-C (mmol/L)	2.19±0.67	1.76±0.35*	1.17±0.18*	1.09±0.26*

SCr (mmol/L)	85.03±61.83	72.49±17.47	79.15±18.87	77.64±19.28
UA (mmol/L)	340.37±97.73	307.22±104.65*	340.49±91.25	330.88±99.33
NTL Plaque Progression (%)	153 (23.79)	9 (23.08)	28 (25.23)	6 (11.11)*
Medications				
DAPT				
Baseline	620 (96.42)	39 (100)*	102 (91.89)*	50 (92.59)
Follow up	521 (81.03)	30 (76.92)	84 (75.68)	40 (74.07)
Statin				
Baseline	623 (96.89)	38 (97.44)	109 (98.20)	54 (100)*
Follow up	623 (96.89)	38 (97.44)	107 (96.40)	54 (100)*
β-blocker				
Baseline	459 (71.38)	27 (69.23)	79 (71.17)	39 (72.22)
Follow up	459 (71.38)	26 (66.67)	72 (64.86)	39 (72.22)
ACEI or ARB				
Baseline	374 (58.16)	19 (48.72)	69 (62.16)	35 (64.81)
Follow up	345 (53.65)	19 (48.72)	61 (54.95)	31 (57.41)

Data are expressed as mean±SD, or frequency counts (percentages), as appropriate. DM, diabetes mellitus; CKD, chronic kidney disease; ACS, acute coronary syndrome; FPG, fasting plasma glucose; HbA1C, glycosylated hemoglobin; TG, triglycerides; TC, total cholesterol; HDL-C, high-density lipoprotein-cholesterol; LDL-C, low-density lipoprotein-cholesterol; SCr, serum creatinine; UA, uremic acid; CAG, coronary angiography; NTL, non-target lesion; DAPT, dual anti-platelet therapy; ACEI, angiotensin-converting enzyme inhibitor; ARB, angiotensin receptor blocker. *$p<0.05$ compared with **Non-optimal Subgroup A**.
